# Inhibiting histone deacetylase 6 partly protects cultured rat cortical neurons from oxygen-glucose deprivation-induced necroptosis

**DOI:** 10.3892/mmr.2015.3779

**Published:** 2015-05-13

**Authors:** LIMING YUAN, ZHEN WANG, LIHUA LIU, XIAOHONG JIAN

**Affiliations:** 1 Department of Anatomy, Medical College of Hunan Normal University, Changsha, Hunan 410013, P.R. China; 2 Department of Anatomy and Neurobiology, Xiangya School of Medicine, Central South University, Changsha, Hunan 410000, P.R. China; 3 Department of Nursing, Medical College of Hunan Normal University, Changsha, Hunan 410013, P.R. China

**Keywords:** histone deacetylase 6, necroptosis, oxygen-glucose deprivation, ischemia, neuron

## Abstract

Necroptosis has an important role in ischemia-reperfusion damage. The expression of histone deacetylase 6 (HDAC6) is upregulated in neurons following ischemia-reperfusion, however, whether HDAC6 is closely involved in the necroptosis, which occurs during ischemia-reperfusion damage remains to be elucidated. In the present study, the roles of HDAC6 in the necroptosis of cultured rat cortical neurons were investigated in a oxygen-glucose deprivation (OGD) model. The results demonstrated that OGD induced marked necroptosis of cultured rat cortical neurons and upregulated the expression of HDAC6 in the cultured neurons, compared with the control (P<0.05). The necroptosis inhibitor, necrostatin-1 (Nec-1), decreased The expression of HDAC6 in the OGD-treated cultured neurons, accompanied by the inhibition of necroptosis. Further investigation revealed that, compared with OGD treatment alone, inhibiting the activity of HDAC6 with tubacin, a specific HDAC6 inhibitor, reduced the OGD-induced necroptosis of the cultured rat cortical neurons (P<0.05), which was similar to the change following treatment with Nec-1 (P>0.05). In addition, inhibiting the activity of HDAC6 reversed the OGD-induced increase of reactive oxygen species (ROS) and the OGD-induced decrease of acetylated tubulin in the cultured rat cortical neurons (P<0.05), compared with the neurons treated with OGD alone). The levels of acetylated tubulin in the cultured neurons following treatment with OGD and tubacin were significantly higher than those in the control (P<0.05). These results suggested that HDAC6 was involved in the necroptosis of neurons during ischemia-reperfusion by modulating the levels of ROS and acetylated tubulin.

## Introduction

Ischemic stroke is a leading cause of disability and mortality worldwide, which is triggered by vascular occlusion via either *in situ* thrombosis or embolization of a clot from a proximal arterial or cardiac source (1–3). The area of the brain, which is reliant exclusively on the blood supply from the occluded vessel is termed the ‘ischemic core’ (4,5). The area surrounding the ischemic core is referred to as the ‘penumbra’, which is perfused, in part, by collateral blood flow (4,5). Usually, vascular occlusion induces the deprivation of glucose and O_2_ and leads to deprivation in the ischemic brain areas, particularly at the ischemic core (4–6). This finally results in the damage of neurons in the ischemic brain areas by initiating a complex cascade of cellular events, including glutamate-induced excitotoxicity, free radical-mediated injury and inflammation (4–8). Therefore, following ischemic stroke, patients usually exhibit motor, sensory or cognitive function, which leads to serious financial burden for the patient and their families (4–8). At present, the most successful therapeutic strategy for ischemic stroke is reperfusion (6,7). However, restoration of perfusion to ischemic brain areas can exacerbate tissue damage by mechanisms, including the generation of reactive oxygen species (ROS) by mitochondria and the increased recruitment of inflammatory cells, (6,7). Thus, inhibiting or reducing the damage caused by reperfusion is an important issue in the treatment of ischemic stroke.

Programmed necrosis, termed, necroptosis was originally reported by Degterev *et al* in 2005 (9). It is a form of cell death, which is distinctly different from necrosis and apoptosis. It is characterized by cell swelling, mitochondria dysfunction, cell membrane permeabilization and the release of cytoplasmic content to the extracellular space (10). However, DNA fragmentation does not occur (10). Degterev *et al* demonstrated that middle cerebral artery occlusion (MCAO) induced significant brain infarction and behavioral defects of mice (9). The necroptosis specific inhibitor, necrostatin-1 (Nec-1), significantly decreased the volume of brain infarction and improved the behavior of mice when administered 2 or 6 h following MCAO (9). Similar results were also detected in cultured hippocampal neurons following oxygen-glucose deprivation (OGD), an ischemia-reperfusion model (11). Inhibiting necroptosis significantly decreases the OGD-induced loss of cultured hippocampal neurons (11), suggesting that necroptosis is important in ischemia-reperfusion damage. Additional studies have revealed that receptor-interacting protein (RIP)1, RIP3 and ROS are important signaling molecules in necropolis (11,12). However, the molecular networks underlying necroptosis during ischemia-reperfusion damage remain to be elucidated.

Histone deacetylase 6 (HDAC6) is a member of the class IIb histone deacetylases and is present predominantly in the cytosol (13,14). HDAC6 is expressed widely in the brain (15). Chen *et al* demonstrated that the expression of HDAC6 is markedly upregulated in the mice cortex at 3 h and 12 h of reperfusion following MCAO (16). This study also demonstrated that the expression of HDAC6 increases in cultured mouse hippocampal neurons following OGD (16). These findings suggested that HDAC6 is important in ischemia-reperfusion damage. Notably, previous studies have revealed that HDAC6 enhances neuronal oxidative stress by deacetylating peroxiredoxin-1 and peroxiredoxin-2 (17). HDAC6 also decreases mitochondrial transport by modulating the acetylation level of α-tubulin (13,18). Mitochondria-associated oxidative stress is the major mechanism of damage in neuron necroptosis during ischemia-reperfusion (6,7). Therefore, the present study hypothesized that HDAC6 is closely involved in the necroptosis of neurons during ischemia-reperfusion. The present study investigated the effects of OGD on the expression of HDAC6 in cultured rat cortical neurons, and its association with the necroptosis of neurons following OGD.

## Materials and methods

### Materials

The primary antibody against HDAC6 (cat. no. PAB8753) was purchased from Abnova (Taibei, Taiwan), antibodies against acetylated (Ac)-tubulin (cat. no. T6793), microtubule associated protein (MAP)2 (cat. no. M9942), necroptosis inhibitor, Nec-1 (cat. no. N-9037) were purchased from Sigma-Aldrich (St. Louis, MO, USA) and the HDAC6 specific inhibitor, tubacin was purchased from ChemieTek (Indianapolis, IN, USA). Propidium iodide (PI) was purchased from Jackson ImmunoResearch Laboratories, Inc. (West Grove, PA, USA). The following secondary antibodies were purchased from Jackson ImmunoResearch, Laboratories, Inc.: Polyclonal Alexa Fluor-488 AffiniPure donkey anti-rabbit IgG (1:500; 711-545-152) and polyclonal Alexa Fluor-594 AffiniPure donkey IgG (1:500; 711-585-152). The Reactive Oxygen Species Assay kit (cat. no. C13293) was purchased from Invitrogen Life Technologies (Shanghai, China).

### Animals

All experimental procedures were approved by the Institutional Review Board of the Third Xiangya Hospital of Central South University (Changsha, China). A total of six pregnant Sprague-Dawley rats (18 days of pregnancy; ~3 months old) were purchased from Central South University. All the rats were raised under controlled environmental conditions on a 12 h light/dark cycle at 22–24°C with *ad libitum* access to food and water and were housed alone.

### Primary neuron culture

Primary cultures of rat cortical neurons were prepared from embryonic day 18 (E18) rats (removed from the pregnant rats), using a previously described procedure (19). Briefly, the cortex of the E18 rat was dissected and washed five times in Hank’s balanced salt solution (HBSS; Invitrogen Life Technologies, Carlsbad, CA, USA). The cortex tissue was then digested using 0.125% trypsin (Invitrogen Life Technologies) for 13 min at 37°C. Following three washes in HBSS, the digested cortex was dissociated from the meninges and subcortical structures and plated on the coverslips coated with poly-D-lysine in Dulbecco’s modified Eagle’s medium (Invitrogen Life Technologies) with 10% fetal bocine serum (Invitrogen Life Technologies) and 1% penicillin-streptomycin (Invitrogen Life Technologies). After 4 h, the cultured medium (37°C) was replaced with neurobasal medium with 1% B27 (Invitrogen Life Technologies). Half of the maintenance medium (neurobasal medium + 1% B27) of THE cultured neurons was replaced every 2 days.

### Oxygen-glucose deprivation (OGD) and drug treatments

OGD was prepared according to previously reported methods (11,20). Briefly, on the fourth day of culture, the cultured cortical neurons were divided into control, OGD, OGD+Nec-1 and OGD+tubacin groups. The neurons in the OGD group were placed in glucose-free deoxygenated buffer medium (OGD medium; Dulbecco’s modified Eagle’s medium without glucose; Gibco Life Technologies, Shanghai, China) with solvent inside an anaerobic OGD chamber with 5% CO_2_ and residual levels of O_2_ (Thermo Forma 1029; Thermo Fisher Scientific, Waltham, MA) at 37°C for 2 h. The neurons in the OGD + Nec-1 group were placed in 2 ml OGD medium with 2 *μ*l 1% Nec-1 (Sigma-Aldrich) (11), inside an OGD chamber with 5% CO_2_ and residual levels of O_2_ at 37°C for 2 h. The neurons in the OGD + tubacin group were placed in OGD medium with 2.5 *μ*M tubacin (21), inside an OGD chamber with 5% CO_2_ and residual levels of O_2_ at 37°C for 2 h. The neurons in the control group were placed in a similar buffer, containing 25 mM glucose (Sigma-Aldrich) and 2 µl dimethyl sulfoxide (DMSO), and maintained for 2 h in a humidified incubator with 5% CO_2_/95% air at 37°C. Following incubation, the neurons of each group were placed in their conditioned medium and returned to the normoxic incubator (Thermo Forma 1029; Thermo Fisher Scientific) for 3 h recovery.

### Nuclear morphology

Neuronal death was assessed by analyzing the nuclear morphology 3 h after OGD. At this time-point, the neurons in each group were stained using the nuclear dye, PI (2 *μ*g/ml), for 8 min at 37°C. The neurons were then washed, fixed in 4% paraformaldehyde (Sigma-Aldrich), washed again with 0.01 M phosphate-buffered saline (PBS) and covered with mounting medium (Vector Laboratories, Inc., Burlingame, CA, USA) and 4′,6-diamidino-2-phenylindole (DAPI; Vector Laboratories, Inc.). The PI staining and chromatin condensation of the neurons in each group were immediately analyzed under fluorescence microscopy (Nikon 80i; Nikon, Tokyo, Japan). According to a method described by Vieira (11), ‘positive PI staining’ was considered a necrotic marker, since membrane leak is one of the predominant features of necrotic cell death (11). Chromatin condensation and pyknosis, revealed by the DAPI staining were referred to as apoptotic markers (11). Necrotic-like neuronal death was marked by positive PI staining (11). The percentages of necrotic cell death (PI-positive cells / total number of cells) and of apoptotic-like cell death (apoptotic-like nuclei / total number of cells) were calculated.

### Immunofluorescence

Following washing with 0.01 M PBS for 10 min three times, the neurons were incubated in blocking solution (Sigma-Aldrich), containing 5% bovine serum albumin and 0.3% Triton X-100 in 0.01 M PBS, for 1 h at room temperature. The neurons were then incubated with primary antibodies (rabbit anti-HDAC6; 1:500; cat. no. PAB8753; Abnova), mouse anti-ac-tubulin (1:1,000; cat. no. T6793; Sigma-Aldrich), mouse anti-MAP2 (1:1000; cat. no. M9942; Sigma-Aldrich) overnight at 4°C. Following incubation, the neurons were washed with 0.01 M PBS three times and then incubated with secondary antibodies labeled with fluorescent dyes (1:500, Jackson ImmunoResearch Laboratories, Inc.) for 2 h at room temperature. Following three washes in PBS, the neurons were covered with mounting medium and DAPI. As negative controls, normal neurons were processed using the same procedures without the primary antibodies. The immunofluorescence intensities of the HDAC6 and Ac-tubulin staining in each group were detected using the Nikon Eclipse 80i microscope and Image J software, version 1.48.

### ROS detection

The level of intracellular ROS was detected using a Reactive Oxygen Species Assay kit, according to a previously reported method (22), in which DCFH-DA (Invitrogen Life Technologies), a fluorescent probe, is oxidized by ROS in viable cells to 2′,7′-dichlorofluorescein. Briefly, the cultured neurons were incubated with 100 *μ*M DCFH-DA dissolved in DMSO for 30 min at 37°C. Following times washes with PBS, the cultured neurons were covered and detected using fluorescence microscopy. The ROS fluorescence intensity was detected using ImageJ software.

### Statistical analysis

Data are presented as the mean ± standard deviation and were analyzed using one-way analysis of varianced followed by a Student-Newman-Keuls test. P<0.05 was considered to indicate a statistically significant difference.

## Results

### OGD upregulates the expression of HDAC6 and induces necroptosis of cultured rat cortical neurons

**Figure 1:**
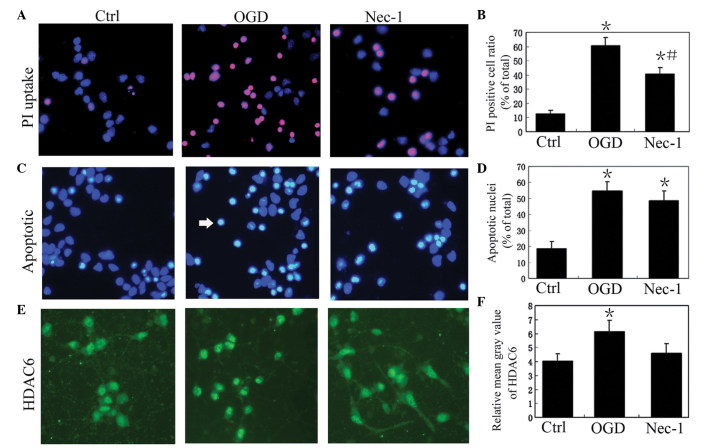
OGD upegulates the expression of HDAC6 and induces the necroptosis of cultured rat cortical neurons. (A) Representative images of PI staining of cultured rat cortical neurons in the normal control (Ctrl), OGD and OGD + Nec-1 groups. Magnification, ×400. (B) PI-positive cell ratio (number of PI-positive cells / total number of cells) in the Ctrl, OGD and Nec-1 groups (n=6). Data are presented as the mean ± standard deviation. ^*^P<0.05, vs. Ctrl; ^#^P<0.05, vs. OGD. (C) Representative images of apoptotic-like cells in the cultured rat cortical neurons in Ctrl, OGD and Nec-1 groups. The white arrow indicates an apoptotic nuclei. DAPI staining; magnification, ×400. (D) Apoptotic-like cell ratio in the Ctrl, OGD and Nec-1 groups (n=6). Data are presented as the mean ± standard deviation. ^*^P<0.05, vs. Ctrl. (E) Representative images of HDAC6 staining of cultured rat cortical neurons in the Ctrl, OGD and Nec-1 groups. HDAC6 immunofluorescence; magnification, ×400. (F) Relative mean gray value of HDAC6 staining in the Ctrl, OGD and Nec-1 groups (n=6). Data are presented as the mean ± standard deviation. ^*^P<0.05, vs. Ctrl. OGD, oxygen-glucose deprivation; HDAC6, histone deacetylase 6; Nec-1, necrostatin-1; PI, propidium iodide.

OGD is a commonly-used *in vitro* model of ischemia-reperfusion. The present study investigated necroptosis and the expression of HDAC6 in cultured rat cortical neurons following OGD. The results revealed that the percentage of necrotic cell death, determined by the number of PI-positive cells without pyknosis / total number of cells, in the OGD group was 60.5±5.8%, which was significantly higher than that of the control (12.5±2.5%) and OGD+Nec-1 (40.8±4.3%) groups (P<0.05; Fig. 1). The percentage of necrotic cell death in the OGD+Nec-1 group (40.8±43%) was significantly higher than that of the control group (P<0.05; Fig. 1). The percentage of apoptotic-like cell death, calculated as the number of apoptotic-like nuclei / total number of cells, in the OGD group (54.7±5.8%) was markedly higher, compared with that of the control group (18.6±4.5%; Fig. 1). However, no significant difference was observed in the percentage of apoptotic-like cell death between the ODG and OGD+Nec-1 (48.6±6.2%) groups (P>0.05; Fig. 1). These results suggested that ODG induced marked necroptosis in The cultured rat cortical neurons, which was inhibited by Nec-1.

In accordance with the higher level of neuron necroptosis in the OGD group, the immunofluorescence intensity of the HDAC6 staining was also increased in the neurons of the OGD group, compared to that of the control group (P<0.05; Fig. 1). In the OGD+Nec-1 group, the immunofluorescence intensity of the HDAC6 staining was lower, compared with that of the OGD group (P<0.05; Fig. 1).

### Inhibiting HDAC6 activity reduces OGD-induced necroptosis in cultured rat cortical neurons

**Figure 2:**
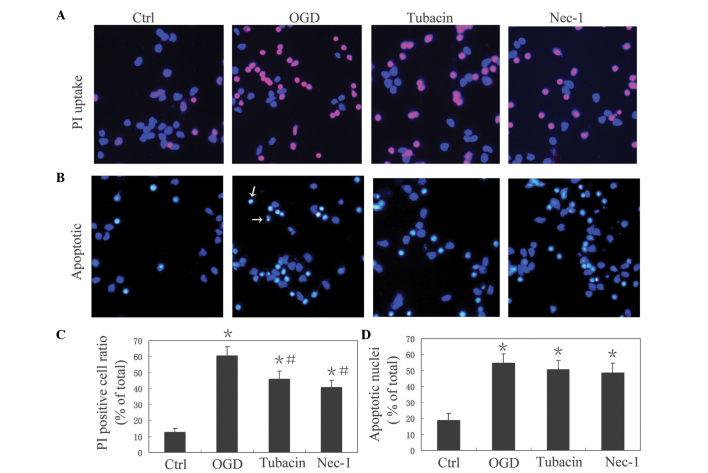
Inhibiting the activity of HDAC6 reduces the OGD-induced necroptosis of cultured rat cortical neurons. (A) Representative images of PI staining of cultured rat cortical neurons in the normal control (Ctrl), OGD, OGD + tubacin and OGD + Nec-1 treatment groups. Magnification, ×400. (B) Representative images of apoptotic-like cells in the Ctrl, OGD, tubacin and Nec-1 groups. The white arrows indicates apoptotic nuclei. Magnification, x400. (C) PI-positive cell ratio (number of PI-positive cells / total number of cells) in the Ctrl, OGD, tubacin and Nec-1 groups (n=6). Data are presented as the mean ±standard deviation. ^*^P<0.05, vs. Ctrl; ^#^P<0.05, vs. OGD. (D) Apoptotic-like cell ratio in the Ctrl, OGD, tubacin and Nec-1 groups (n=6). Data are presented as the mean ±standard deviation. ^*^P<0.05, vs. Ctrl. OGD, oxygen-glucose deprivation; HDAC6, histone deacetylase 6; Nec-1, necrostatin-1; PI, propidium iodide.

In order to investigate the role of HDAC6 in the OGD-induced necroptosis of cultured rat cortical neurons, the present study compared the OGD-induced necroptosis of cultured rat cortical neurons with and without the HDAC6 specific inhibitor, Tubacin. The percentage of necrotic cell death in the OGD+Tubacin group was 45.8±5.3%, which was significantly lower than that of OGD group (60.5±5.8%; P<0.05), but was not significantly different to that of the OGD+Nec-1 group (40.8±4.3%; P>0.05; Fig. 2). The percentage of apoptotic-like cell death in the OGD+Tubacin group was 50.6±5.7%, which was similar to that of the OGD group (547±5.8%; P>0.05) and OGD+Nec-1 group (48.6±6.2%; P>0.05; Fig. 2). These results suggested that inhibiting HDAC6 activity reduced the OGD-induced necroptosis of cultured rat cortical neurons.

### Inhibiting HDAC6 activity decreases the level of ROS and increases the level of acetylated tubulin in cultured rat cortical neurons following OGD treatment

**Figure 3:**
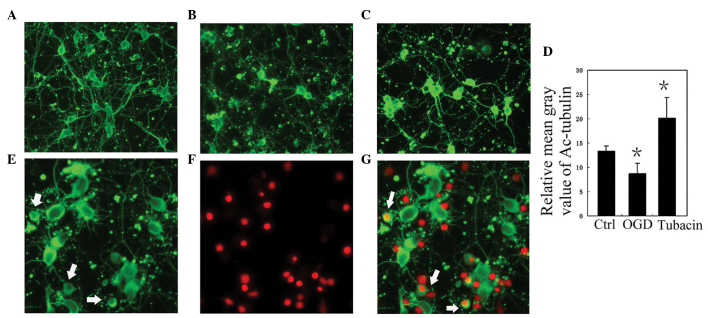
Inhibiting the activity of HDAC6 increases the level of acetylated tubulin in cultured rat cortical neurons following OGD treatment. (A–C) Representative images of Ac-tubulin staining of cultured rat cortical neurons in the (A) normal control (Ctrl), (B) OGD and (C) OGD + tubacin treatment groups. Magnification, x400. (D) Analysis of the relative mean gray value of Ac-tubulin staining in the Ctrl, OGD, and tubacin groups. Data are presented as the mean ±standard deviation. *P<0.05, vs. Ctrl. (E-G) Co-staining of Ac-tubulin (green) and PI (red), at the same visual field, in the OGD group. The Ac-tubulin-positive processes of the PI-positive neurons disappeared (white arrows). Magnification, ×600. OGD, oxygen-glucose deprivation; HDAC6, histone deacetylase 6; PI, propidium iodide; Ac, acetylated.

**Figure 4:**
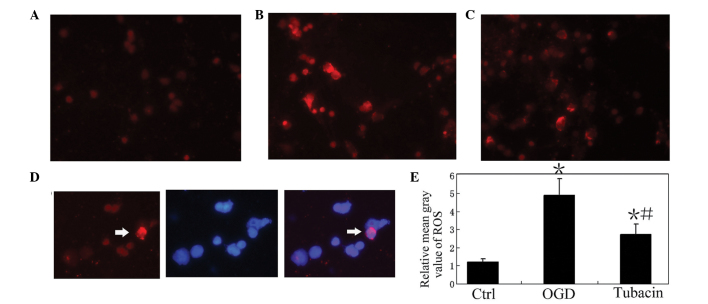
Inhibiting the activity of HDAC6 decreases the level of ROS in cultured rat cortical neurons following OGD treatment. (A–C) Representative images of ROS staining of cultured rat cortical neurons in the (A) control (Ctrl), (B) OGD treatment and (C) OGD + tubacin treatment groups. Magnification, ×400). (D) Co-staining of ROS (red) and 4′,6-diamidino-2-phenylindole (blue), at the same visual field, in the OGD group. The white arrow indicates an apoptotic-like cell with a high level of ROS. Magnification, ×600. (E) Analysis of the relative mean gray value of ROS staining in the Ctrl, OGD and Tubacin groups. Data are presented as the mean ±standard deviation. ^*^P<0.05, vs. Ctrl; ^#^P<0.05, vs. OGD. HDAC6, histone deacetylase 6; ROS, reactive oxygen species.

Mitochondria-associated ROS are important in the necroptosis of neurons during ischemia-reperfusion. It is now established that HDAC6 is central in neuronal oxidative stress and in mitochondrial transport. Acetylated tubulin is closely involved in mitochondrial transport, therefore, the present study examined the levels of ROS and acetylated tubulin in each group. Compared with the control group, the level of acetylated tubulin in the OGD group was reduced (P<0.05; Fig. 3). Notably, the positive processes of acetylated tubulin in the PI-positive neurons disappeared (Fig. 3). By contrast, the level of acetylated tubulin in the OGD+tubacin group increased significantly, compared with that of the OGD group (P<0.05; Fig. 3). Compared with the control, the level of ROS in the OGD group was significantly increased (P<0.05; Fig. 4). Following the inhibition of HDAC6 activity, the level of ROS in the OGD+Tubacin group was significantly lower than that of the OGD group (P<0.05; Fig. 4).

## Discussion

The aim of the present study was to investigate whether HDAC6 is involved in the necroptosis of neurons during ischemia-reperfusion. The results demonstrated that OGD induced the necroptosis of cultured rat cortical neurons, accompanied by an increase in the expression of HDAC6. Inhibiting the activity of HDAC6 reduced the necroptosis of the cultured rat cortical neurons following OGD treatment. In addition, inhibiting the activity of HDAC6 also decreased the level of ROS and increased the level of acetylated tubulin in the cultured rat cortical neurons following OGD treatment. These results suggested that HDAC6 was involved in the necroptosis of neurons during ischemia-reperfusion by modulating the levels of ROS and acetylated tubulin.

Multiple cellular processes are rapidly activated during ischemic-reperfusion. At the ischemic core, artery occlusion leads to rapid energy depletion and results in the necrosis of neurons. However, in the ischemic penumbra, metabolism and intracellular signaling cascades are maintained partly by a collateral blood supply during the ischemia (23). Therefore, neurons in the ischemic penumbra are usually damaged by necroptosis or apoptosis. Notably, Xu *et al* demonstrated that the specific necroptosis inhibitor, Nec-1, significantly reduced the volume of infarction between 59.3±2.6 and 47.1±1.5% in mice with MCAO/reperfusion (23). In addition, the neuroprotective effect of Nec-1 remained when it was used 6 h after MCAO/reperfusion (9). These results revealed that anti- necroptosis therapy is of benefit following ischemic stroke. In the present study, OGD also induced the necroptosis of cultured rat cortical neurons and upregulated the expression of HDAC6 in the cultured rat cortical neurons (Fig. 1). These observations were consistent with previous reports. Vieira *et al* demonstrated that OGD for 2 h leads to a marked loss of cultured neurons by necroptosis, and Chen *et al* observed that the expression of HDAC6 in neurons was markedly upregulated following OGD treatment or MCAO (16). In order to determine the role of HDAC6 in the OGD-induced necroptosis of cultured rat cortical neurons, the present study exposed the neurons to tubacin, HDAC6 specific inhibitor, and detected the damage of neurons. The results demonstrated that, similar to the effects of Nec-1, tubacin significantly decreased the necrotic neuron death induced by OGD, but had no significant affect on apoptotic-like neuron death. It is well known that necrosis cannot be modulated, however, necroptosis can. Thus, tubacin reduced OGD-induced necrotic neuron death, possibly by inhibiting necroptosis of the neurons. This suggested that HDAC6 may modulate the OGD-induced necroptosis of cultured rat cortical neurons. Further investigations revealed that inhibiting the activity of HDAC6 reversed the increased levels of ROS and the decreased levels of acetylated tubulin in the OGD-treated neurons. Previous studies demonstrated that mitochondria-associated oxidative stress is a major mechanism of damage in neuron necroptosis (23), and the acetylation level of α-tubulin modulates mitochondrial transport (24). Thus, HDAC6 may function in OGD-induced necroptosis of cultured rat cortical neurons by modulating the levels of ROS and acetylated tubulin. However, the signaling pathway of HDAC6 in the modulation of necroptosis remains to be elucidated. Parmigiani *et al* (17) observed that HDAC6 enhances neuronal oxidative stress by deacetylating peroxiredoxin-1 and peroxiredoxin-2. In addition, Rip1 and Rip3 are key molecules in necroptosis (25) and may be direct or indirect targets of HDAC6. Collectively, the results of the present study demonstrated that HDAC6 is an important molecule in the necroptosis of neurons during ischemia-reperfusion, and additionally suggest HDAC6 as a possible therapeutic target for the protection of neurons during ischemia-reperfusion.
